# Blood Donor Serological Screening in Makkah, Saudi Arabia: A 7-Year Retrospective Study on Transfusion-Transmitted Infections

**DOI:** 10.1155/ijm/3257549

**Published:** 2025-07-07

**Authors:** Amr J. Halawani, Afnan J. Hawsawi, Latifa A. Jaber, Saeed M. Kabrah, Turki M. Maghrabi, Khaled M. Alobaid, Alaa A. Kaki, Ayman M. Aljabri, Adel S. Alsaedi, Mohammed M. Alharbi, Abdullah F. Alhazmi, Hani A. Alsaedi, Ahmed S. Almalki, Fatima A. Alharthi, Yahya H. Almalki, Abdullah M. Alotaibi, Rakan M. Abu-Harba, Ahmad F. Arbaeen, Hesham A. Malak, Mohammad O. Sabbag, Rani O. Alnabati

**Affiliations:** ^1^Department of Clinical Laboratory Sciences, Faculty of Applied Medical Sciences, Umm Al-Qura University, Makkah, Saudi Arabia; ^2^Department of Laboratory and Blood Bank, King Abdulaziz Hospital, Ministry of Health, Makkah, Saudi Arabia; ^3^Department of Biology, Faculty of Applied Science, Umm Al-Qura University, Makkah, Saudi Arabia; ^4^Central Blood Bank Department, Health Affairs in Makkah Region, Saudi Arabia Ministry of Health (MOH), Makkah, Saudi Arabia

**Keywords:** blood donors, blood transfusion, infectious diseases, Saudi Arabia, transfusion-transmitted infections

## Abstract

**Introduction:** Screening donated blood for transfusion-transmitted infections (TTIs) is a critical component of transfusion safety, particularly in high-demand regions such as Makkah City, Saudi Arabia. This study is aimed at assessing the seroprevalence of TTI markers among blood donors at King Abdulaziz Hospital-Makkah (KAHM) over a 7-year period.

**Methods:** In this retrospective study, 17,661 individuals who donated blood at the KAHM, Saudi Arabia, from January 2017 to December 2023, were included. The prevalence of TTI markers was assessed and categorized by year, gender, age, type of donors (whole blood and apheresis), and category of donation (replacement and volunteer). In addition to ABO group testing, commercially available kits were used for serological tests.

**Results:** Among donors, 74 (0.42%) were reactive for HBsAg, 1419 (8.03%) for HBcAb, and 1295 (7.33%) for HBsAb. Other reactive cases included HCV (0.29%), HIV (0.06%), HTLV-I/II (0.05%), and syphilis (0.44%). No cases of malaria were reported. Statistically significant variations were observed across years for HBsAg (*p* = 0.007), HBsAb, and HBcAb (*p* < 0.001), suggesting changes in infection exposure, immunity, or donor screening efficiency. Male donors represented 97.17% of the cohort, and replacement donors were more prevalent (54.75%) than volunteers.

**Conclusion:** The overall prevalence of TTIs among blood donors in Makkah was low, affirming the effectiveness of current screening protocols. However, the relatively high prevalence of hepatitis B core antibodies indicates prior exposure among a notable proportion of donors. Yearly fluctuations in seropositivity suggest evolving epidemiological patterns, warranting continuous surveillance and targeted public health interventions.

## 1. Introduction

Blood donation is a method of drawing blood from a healthy donor, collecting and storing it for transfusion to patients with matching blood types. Blood transfusion is a therapeutic procedure [1]. It is estimated that about 92 million people donate blood yearly [2]. Nevertheless, the prevalence of clinically transfusion-transmitted infections (TTIs) places a significant concern on the healthcare system. TTIs involve infections transmitted from person to person through parenteral administration of infected blood or blood products [3].

Several infections are classified as blood transfusion infections. Most widespread transfusion-associated risks worldwide include hepatitis B virus (HBV), human immunodeficiency virus (HIV), hepatitis C virus (HCV), human T-cell lymphotropic virus (HTLV), malaria, and syphilis. These infections can cause both acute and chronic diseases. Mainly, HIV, HBV, and HCV are considered threats to blood safety because of the constant incidence in the blood as a carrier or latent state [3]. Therefore, donor selection is essential since the infected individual acts as an asymptomatic reservoir and a possible transmission resource [4]. Furthermore, from an economic and human viewpoint, an unsafe blood transfusion is highly costly for the recipients and not only for the recipients but also for their families and communities [5].

However, clinically significant TTIs have been markedly reduced over the past decades. These reductions have occurred due to the development and improvement in blood and blood component collection, processing, and screening procedures [6]. Moreover, the World Health Organization (WHO) recommends that each country develop a national blood policy as part of its national health policy. In addition, they must create and manage blood transfusion facilities by providing sufficient and safe blood and blood products and making them accessible to meet the transfusion needs of the population [7, 8].

In Saudi Arabia, several regional studies have investigated the prevalence of TTIs among blood donors. Alqahtani et al. reported the seroprevalence of HBV and HCV in the northern region of Riyadh Province [1], while Alabdulmonem et al. examined the association between ABO and Rh blood groups and TTI markers among donors in the central region [3]. More recently, Minshawi et al. conducted a large-scale study in Makkah, identifying regional patterns of infection and supporting the need for localized transfusion safety strategies [8]. Despite these efforts, variations in donor demographics, religious tourism, and seasonal demand (especially during Hajj and Umrah) make Makkah a unique context for studying TTIs [9].

Screening of blood products is a required protocol implemented in healthcare facilities to reduce the onset of TTIs [3]. Donor screening includes the selection of low-risk blood donors by using a donor history questionnaire, predonation physical examination, and initial rapid donor testing. On the other hand, donor testing includes direct pathogen detection and serological tests. Moreover, donor testing was performed again after donation to ensure blood safety. Serological testing is the standard screening method for blood donations. Immunoassays detect antibodies against viruses or viral antigens. Immunoassays involve a “window period” (WP) between the donor's virus exposure and the production of antibodies against it. During this interval, immunoassay testing may overlook infections in given blood. A large percentage of TTI cases result from undetected WP infections. This period might outcome in false-negative results. Therefore, serological and molecular method approaches may be necessary to raise the sensitivity [10]. Nucleic acid testing (NAT) regulates viral dynamics and ensures safe blood donation.

Evaluating trends in the prevalence of TTIs among blood donors is essential for estimating the effectiveness of blood safety strategies [8, 11]. Also, it gives clues to health policy makers to improve the existing blood bank strategies to reduce the potential risk of acquiring these infections through blood transfusion. In this study, we aimed to collect and analyze the demographic data of blood donors in Makkah City, Saudi Arabia, from 1 January 2017 to December 2023; determine the prevalence of all tested TTI markers among blood donors utilizing data from serology assays; and classify the “reactive” donations for serological assays according to donor characteristics such as sex, age, donation type, and blood group.

## 2. Materials and Methods

### 2.1. Study Design

A retrospective cross-sectional study was conducted using the records of blood donors from the Blood Bank Centre at King Abdulaziz Hospital-Makkah (KAHM), covering the period from January 2017 to December 2023.

### 2.2. Ethics Statement

The study received ethical approval from the Institutional Review Board (IRB) in Makkah under Reference Number H-02-K-076-0622-759. Given the retrospective nature of the study, informed consent was not required, as only donor records were utilized, and no direct contact with donors was made.

### 2.3. Data Access and Retrieval

Before data retrieval, formal administrative approval was obtained from the hospital's Department of Blood Bank Services and the Medical Records Department. A data access request was submitted, and upon approval, authorized personnel from the research team were granted permission to access the secure hospital information system. Data were retrieved from the hospital's electronic donor management system using a standardized extraction form. This included demographic variables (age, gender, and nationality), donation details (volunteer or replacement), blood group, Rh type, and serological results for TTIs.

To ensure confidentiality, all identifying personal information was excluded, and a unique anonymous code was assigned to each record. Extracted data were verified by cross-referencing with monthly TTI surveillance logs maintained by the blood bank. The cleaned dataset was exported into Microsoft Excel and subsequently imported into SPSS (Version 28, IBM Corp.) for statistical analysis.

### 2.4. Study Participants

Included in the study were all donors who met the eligibility criteria set by the Saudi Ministry of Health: age 17–65 years, body weight ≥ 50 kg, hemoglobin levels of 14–17 g/dL for males and 12–14 g/dL for females, pulse rate 50–100 bpm, body temperature not exceeding 37°C, and blood pressure below 120/80 mm Hg. Only complete records from successful donations that included full serological data for TTIs were analyzed. Incomplete or ineligible donations were excluded.

### 2.5. Laboratory Tests

All blood donations were subjected to both serological and molecular screening to detect TTIs, following the protocols implemented by the Blood Bank Centre at King Abdulaziz Hospital. Serological testing was performed using commercially available, validated kits.

Two assays were used for HBV screening. The detection of hepatitis B surface antigen (HBsAg) was carried out using the Monolisa HBs Ag ULTRA kit (Bio-Rad Laboratories), with a reported sensitivity of 100% and specificity of 99.9%. In addition, anti-HBV core (HBc) IgG antibodies were tested as part of the HBV panel using the same manufacturer's platform. HCV antibodies were identified using the Monolisa HCV Ag-Ab ULTRA (second-generation kit, Bio-Rad Laboratories), which detects anti-HCV IgG antibodies with a sensitivity of 100% and specificity of 99.9%. Screening for HIV was performed using the Genscreen ULTRA HIV Ag-Ab (fourth-generation kit, Bio-Rad Laboratories), designed to detect both HIV-1/2 antibodies and HIV p24 antigen. This test exhibits a sensitivity and specificity of 100% and 99.9%, respectively. For HTLV Types I and II (HTLV-I/II), the Architect rHTLV-I/II assay (Abbott Diagnostics) was used. This assay targets anti-HTLV-1/2 IgG antibodies and has a sensitivity of 100% and a specificity of 99.5%. Syphilis screening was conducted using a latex agglutination method with the Spinreact kit, which provides both 100% sensitivity and specificity for the detection of treponemal antibodies. Malaria was screened using the Quick Profile Malaria Pf/Pan Antigen test, a rapid immunochromatographic assay that detects *Plasmodium falciparum*–specific and pan-malarial antigens. The test reports a sensitivity and specificity of 100%.

All reactive samples were retested for confirmation, and only confirmed reactive cases were classified as positive for the respective infection. These combined serological and molecular testing strategies ensured comprehensive TTI screening, in alignment with international standards for transfusion safety.

### 2.6. Statistical Analysis

The prevalence of serological markers for TTIs was calculated and presented as percentages to facilitate comparisons between reactive and nonreactive cases across the study period. Chi-square tests were employed to assess statistical significance, with *p* values < 0.05 considered statistically significant and *p* values < 0.01 regarded as highly significant. Statistical analyses were conducted using SPSS software (IBM, Version 28), adhering to the standard procedures for retrospective studies.

## 3. Results

### 3.1. Demographic Characteristics of Blood Donors

Table 1 presents the demographic characteristics of the blood donors included in this study (*n* = 17,661). The vast majority of donors were male, comprising 97.17% (*n* = 17,162), while female donors accounted for only 2.83% (*n* = 499). In terms of nationality, 55.65% (*n* = 9829) were Saudi nationals, whereas 44.35% (*n* = 7832) were of non-Saudi origin. Regarding the type of donation, 54.75% (*n* = 9670) of donations were categorized as replacement donations, while the remaining 45.25% (*n* = 7991) were volunteer donations.

Age distribution analysis showed that donors were predominantly in the 17–30 age group, representing 43.70% (*n* = 7718) of the total sample. Donors aged 31–40 comprised 34.57% (*n* = 6105), followed by 41–50 years at 16.00% (*n* = 2826), 51–60 years at 5.17% (*n* = 913), and over 60 years at 0.56% (*n* = 99).

The distribution of ABO blood groups among donors revealed that the majority were Blood Group O (47.17%, *n* = 8331), followed by Blood Group A (28.71%, *n* = 5071), Blood Group B (18.95%, *n* = 3347), and Blood Group AB (5.16%, *n* = 912). In terms of Rh factor, 91.14% (*n* = 16,096) of the donors were Rh positive, while 8.86% (*n* = 1565) were Rh negative.

#### 3.1.1. Prevalence of Reactive Serological Markers in Blood Donors

Table 2 illustrates the total frequency and percentage of reactive serological markers detected among blood donors at King Abdulaziz Hospital Blood Bank from 2017 to 2023. During this period, a total of 2935 reactive cases were identified across various markers for TTIs.

The most prevalent reactive marker was hepatitis B core antibody (HBcAb), observed in 8.03% of donors (*n* = 1419). This was followed by hepatitis B surface antibody (HBsAb), with a reactivity rate of 7.33% (*n* = 1295). HBsAg was detected in 0.42% of cases (*n* = 74), indicating a lower frequency of active hepatitis B infection compared to antibody markers.

Other TTIs with detectable reactive markers included anti-HCV, present in 0.29% (*n* = 51) of donors, and HIV and HIV P24 antigens, which were found in 0.06% (*n* = 11) of donors. The serological test for syphilis (VDRL) yielded positive results in 0.44% (*n* = 77) of cases. HTLV-I/II was found in 0.05% (*n* = 8) of donors, while no cases of malaria were detected in the donor pool.

#### 3.1.2. Annual Prevalence of Reactive and Nonreactive Serological Markers

**Figure 1:**
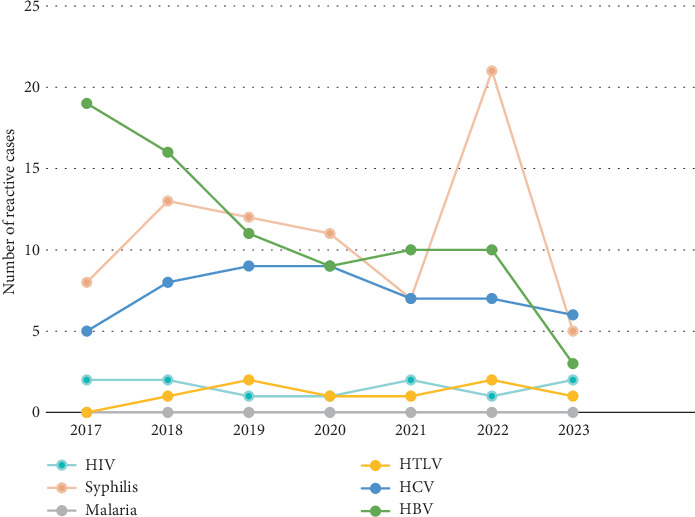
Number of reactive cases for transfusion-transmitted infection serological marker during the study period (2017–2023).

Table 3 provides the annual distribution of reactive and nonreactive serological markers for TTIs among blood donors from 2017 to 2023; the trends were also illustrated in Figure 1.

Over the study period, HBsAg showed a statistically significant decreasing trend in reactivity, with an overall reactivity rate of 0.42% (*n* = 74 and *p* = 0.007). In 2017, the HBsAg reactivity rate was highest at 0.80% (*n* = 19), but it gradually decreased to 0.19% (*n* = 3) by 2023. HBsAb had a statistically significant variation across years (*p* ≤ 0.001), with an overall prevalence of 7.33% (*n* = 1295). The reactivity was highest in 2019, at 9.50% (*n* = 245), and lowest in 2023, at 4.13% (*n* = 66). HBcAb, the most prevalent marker, was detected in 8.03% of donors (*n* = 1419 and *p* ≤ 0.001), with the highest annual rate of 9.69% (*n* = 242) in 2018 and the lowest of 4.88% (*n* = 78) in 2023. For anti-HCV, there was no significant variation across the years (*p* = 0.921), with an overall prevalence of 0.29% (*n* = 51). The highest annual reactivity for anti-HCV was recorded in 2018 at 0.35% (*n* = 9), and the lowest was in 2017 at 0.21% (*n* = 5). Similarly, HIV and HIV P24 antigen reactivity rates remained low and stable (*p* = 0.892), with an overall prevalence of 0.06% (*n* = 11), peaking at 0.13% (*n* = 2) in 2023. The syphilis marker (VDRL) showed a small but nonsignificant variation over time (*p* = 0.181), with an overall reactivity rate of 0.44% (*n* = 77). The highest reactivity rate was 0.70% (*n* = 21) in 2022. Malaria showed no reactivity across all years, as no donors tested positive. HTLV 1 and 2 antibodies were reactive in only 0.05% of donors (*n* = 8) over the study period, showing no significant annual variation (*p* = 0.900). The highest reactivity was in 2018, with 0.08% (*n* = 2), while no reactivity was observed in 2017.

## 4. Discussion

This study provides an in-depth analysis of the prevalence of TTIs among blood donors in Makkah City, Saudi Arabia, from 2017 to 2023. Focusing on HBsAg, HBsAb, HBcAb, anti-HCV, HIV, syphilis, HTLV antibodies, and malaria, the findings offer valuable insights into the patterns of these infections within a key region for religious tourism.

The statistically significant variation in HBsAg prevalence (*p* = 0.007) over the study period suggests potential shifts in hepatitis B infection rates or improvements in donor screening and vaccination strategies. The observed decrease in HBsAg reactivity from 0.80% in 2017 to 0.19% in 2023 may indicate enhanced screening protocols or reduced transmission within the donor population. Similarly, the highly significant *p* values for HBsAb and HBcAb (both *p* ≤ 0.001) may reflect fluctuating immunity and exposure rates among donors. These markers are crucial indicators of past hepatitis B infection and vaccination, highlighting the importance of understanding donor immunity to maintain a safe blood supply, particularly during high-demand periods such as Hajj and Umrah, where donation needs increase substantially [9].

The prevalence of anti-HCV was relatively low (0.29%) in this study, similar to the rates observed previously in Jazan [12] and Makkah [8]. This consistency suggests sustained national efforts to reduce hepatitis C transmission through robust public health strategies and donor deferral policies. Comparatively, other countries, such as Brazil, have reported a much higher HCV prevalence of 2.5%, highlighting the relative success of HCV screening in Saudi Arabia [13]. The low prevalence of HIV (0.06%) is consistent with regional and international studies [8, 14], underscoring effective screening measures and low transmission rates within the donor population. Syphilis reactivity was observed in 0.44% of donors, similar to the prevalence reported by Wanni et al. [15] in the Eastern Province (0.45%), indicating a consistent prevalence across different regions of Saudi Arabia [16]. HTLV antibody reactivity was low in Makkah (0.05%), with slightly higher rates observed in Jeddah (0.08%) [16]. No malaria cases were detected in this study, supporting the effectiveness of travel history screening and the low endemicity of malaria in Saudi Arabia.

From a broader international perspective, this study contributes to global transfusion safety literature by confirming that rigorous screening strategies, when applied consistently, can result in significantly lower TTI prevalence rates, even in settings of high population movement such as Makkah. When compared with data from neighboring countries, it becomes evident that Saudi Arabia has generally lower prevalence rates for TTIs among blood donors. For example, in India, the highest reported prevalence is for HBV (2.1%) [17], while Ethiopia reports a 3.9% HBV prevalence [18]. The states underscore the importance of sustained vaccination efforts in Saudi Arabia to prevent potential transmission through blood donations, particularly in Makkah, where diverse populations converge due to religious tourism. These findings may further enhance donor trust and willingness to become regular, repeat donors, a key element in ensuring a reliable blood supply [19].

The high proportion of male donors (97.17%) is notable, reflecting cultural factors and societal norms in Saudi Arabia. Previous studies have highlighted that female donors in Saudi Arabia face barriers such as medical ineligibility, pregnancy, and cultural beliefs regarding menstruation, which may discourage donation [20]. This gender imbalance, combined with the predominance of replacement (65%), suggests a need for awareness campaigns to encourage voluntary blood donation, particularly among women, to enhance blood supply diversity [9, 19]. Increasing voluntary donation rates not only improves supply stability but may also reduce the risk of TTIs, as voluntary donors are often at lower risk of infection.

The study reveals a substantial reliance on male donors and replacement donations, highlighting a unique gender and donor-type distribution within Saudi Arabia, shaped by cultural and social factors. This pattern suggests an opportunity to enhance voluntary blood donation campaigns, particularly among women, to diversify the donor pool and address gender-related disparities. The low prevalence of other TTIs, such as HCV, HIV, and syphilis, aligns with regional data and reflects the effectiveness of the current screening protocols, which help maintain a safe blood supply essential to Makkah's healthcare needs, particularly during peak pilgrimage seasons when the demand for blood can increase.

Despite its valuable findings, this study has some limitations. Data were collected from a single blood bank at King Abdulaziz Hospital, which may restrict the generalizability of the findings to other regions within Saudi Arabia. Additionally, some donors were excluded due to incomplete records, potentially introducing bias and affecting prevalence rates. A multicenter study across different regions in Saudi Arabia could provide a broader perspective on TTI prevalence and better inform nationwide blood donation policies. Moreover, future studies could integrate molecular testing data, such as NAT results, in routine surveillance to better characterize WP infections and enhance transfusion safety. Additionally, addressing data gaps caused by incomplete records would improve the accuracy of prevalence estimates and offer more detailed insights into TTI transmission dynamics among different demographic groups.

## 5. Conclusion

This study provides a comprehensive evaluation of TTIs among blood donors at KAHM, Saudi Arabia, over a 7-year period from 2017 to 2023. Through the analysis of 17,661 donor records, it offers valuable epidemiological insights into the seroprevalence of key TTIs, including HBV markers, HCV, HIV, HTLV, syphilis, and malaria. The findings confirm a generally low prevalence of TTIs in this donor population, with HBV-related markers being the most detected. These results support the continued effectiveness of the existing donor selection criteria and blood screening protocols employed in Saudi Arabia.

The study meets its objective of monitoring TTI trends and identifying reactive cases across various donor demographics, including age, gender, nationality, donation type, and blood group. This data contributes to national efforts to enhance transfusion safety and informs future public health planning, particularly in a city like Makkah that sees large transient populations due to religious pilgrimages.

Based on these findings, several strategies are recommended to further mitigate TTI risks. Firstly, expanding the scope of surveillance to include multiple blood donation centers across different regions of the Kingdom would enhance the generalizability of future research and offer a more comprehensive national overview. Secondly, promoting voluntary blood donation campaigns, especially among underrepresented groups such as women, could diversify the donor pool and potentially reduce reliance on replacement donations. Standardizing molecular testing, such as NAT, across all centers may improve early detection of WP infections and further strengthen the safety net.

Finally, continued investment in public health education, hepatitis B vaccination programs, and targeted outreach during high-demand seasons such as Hajj and Umrah will be essential to maintaining a secure and reliable blood supply in Makkah. Together, these efforts can ensure that transfusion services remain both safe and responsive to the needs of a rapidly evolving and diverse population.

## Figures and Tables

**Table 1:** Demographic characteristics of blood donors at King Abdulaziz Hospital Blood Bank, Makkah (*n* = 17,661).

**Demographic data**			
		**n**	**%**
Gender	Male	17,162	97.17
	Female	499	2.83

Nationality	Saudi	9829	55.65
	Non-Saudi	7832	44.35

Donation type	Replacement	9670	54.75
	Volunteer	7991	45.25

Age	17–30	7718	43.70
	31–40	6105	34.57
	41–50	2826	16.00
	51–60	913	5.17
	> 60	99	0.56

ABO blood group	O	8331	47.17
	A	5071	28.71
	B	3347	18.95
	AB	912	5.16

Rh type	Positive	16,096	91.14
	Negative	1565	8.86

Number of donors		17,661	100

**Table 2:** Frequency and prevalence of reactive serological markers among blood donors at King Abdulaziz Hospital Blood Bank, Makkah (2017–2023).

**Serological marker**	**Total**	
	**n**	**%**
HBsAg	74	0.42
HBsAb	1295	7.33
HBcAb	1419	8.03
Anti-HCV	51	0.29
HIV and HIV P24	11	0.06
VDRL	77	0.44
Malaria	0	0.00
HTLV 1 and 2	8	0.05
Total	2935	

**Table 3:** Annual frequencies and percentages of reactive and nonreactive serological markers among blood donors at King Abdulaziz Hospital Blood Bank, Makkah (2017–2023).

**Marker/year**		**2017**		**2018**		**2019**		**2020**		**2021**		**2022**		**2023**		**Total**		**p** ** value**
		**n**	**%**	**n**	**%**	**n**	**%**	**n**	**%**	**n**	**%**	**n**	**%**	**n**	**%**	**n**	**%**	
HBsAg	Reactive	19	0.80	16	0.64	11	0.43	9	0.33	10	0.35	6	0.20	3	0.19	74	0.42	0.007⁣^∗^
	Nonreactive	2363	99.20	2482	99.36	2569	99.57	2742	99.67	2842	99.65	2993	99.80	1596	99.81	17,587	99.58	
HBsAb	Reactive	155	6.51	213	8.53	245	9.50	200	7.27	234	8.20	182	6.07	66	4.13	1295	7.33	≤ 0.001⁣^∗^
	Nonreactive	2227	93.49	2285	91.47	2335	90.50	2551	92.73	2618	91.80	2817	93.93	1533	95.87	16,366	92.67	
HBcAb	Reactive	217	9.11	242	9.69	231	8.95	209	7.60	241	8.45	201	6.70	78	4.88	1419	8.03	≤ 0.001⁣^∗^
	Nonreactive	2165	90.89	2256	90.31	2349	91.05	2542	92.40	2611	91.55	2798	93.30	1521	95.12	16,242	91.97	
Anti-HCV	Reactive	5	0.21	8	0.32	9	0.35	9	0.33	7	0.25	7	0.23	6	0.38	51	0.29	0.921
	Nonreactive	2377	99.79	2490	99.68	2571	99.65	2742	99.67	2845	99.75	2992	99.77	1593	99.62	17,610	99.71	
HIV and HIV P24	Reactive	2	0.08	2	0.08	1	0.04	1	0.04	2	0.07	1	0.03	2	0.13	11	0.06	0.892
	Nonreactive	2380	99.92	2496	99.92	2579	99.96	2750	99.96	2850	99.93	2998	99.97	1597	99.87	17,650	99.94	
VDRL	Reactive	8	0.34	13	0.52	12	0.47	11	0.40	7	0.25	21	0.70	5	0.31	77	0.44	0.181
	Nonreactive	2374	99.66	2485	99.48	2568	99.53	2740	99.60	2845	99.75	2978	99.30	1594	99.69	17,584	99.56	
Malaria	Reactive	0	0.00	0	0.00	0	0.00	0	0.00	0	0.00	0	0.00	0	0.00	0	0.00	—
	Nonreactive	2382	100	2498	100	2580	100	2751	100	2852	100	2999	100	1599	100.00	17,661	100.00	
HTLV 1 and 2	Reactive	0	0.00	1	0.04	2	0.08	1	0.04	1	0.04	2	0.07	1	0.06	8	0.05	0.90
	Nonreactive	2382	100	2497	99.96	2578	99.92	2750	99.96	2851	99.96	2997	99.93	1598	99.94	17,653	99.95	
	Total	2382		2498		2580		2751		2852		2999		1599		17,661		

⁣^∗^Highly significant.

## Data Availability

The data that support the findings of this study are available on request from the corresponding author. The data are not publicly available due to privacy or ethical restrictions.
